# Micro-RNA and Proteomic Profiles of Plasma-Derived Exosomes from Irradiated Mice Reveal Molecular Changes Preventing Apoptosis in Neonatal Cerebellum

**DOI:** 10.3390/ijms23042169

**Published:** 2022-02-16

**Authors:** Simonetta Pazzaglia, Barbara Tanno, Ilaria De Stefano, Paola Giardullo, Simona Leonardi, Caterina Merla, Gabriele Babini, Seda Tuncay Cagatay, Ammar Mayah, Munira Kadhim, Fiona M. Lyng, Christine von Toerne, Zohaib N. Khan, Prabal Subedi, Soile Tapio, Anna Saran, Mariateresa Mancuso

**Affiliations:** 1 Laboratory of Biomedical Technologies, Agenzia Nazionale per le Nuove Tecnologie, l’Energia e lo Sviluppo Economico Sostenibile (ENEA), 00123 Rome, Italy; barbara.tanno@enea.it (B.T.); ilaria.destefano@enea.it (I.D.S.); paola.giardullo@enea.it (P.G.); simona.leonardi@enea.it (S.L.); caterina.merla@enea.it (C.M.); annasaran60@gmail.com (A.S.); 2 Department of Woman and Child Health and Public Health, Fondazione Policlinico Universitario A. Gemelli, Istituto di Ricovero e Cura a Carattere Scientifico (IRCCS), 00168 Rome, Italy; gabriele.babini@guest.policlinicogemelli.it; 3 Department of Biological and Medical Sciences, Oxford Brookes University, Oxford OX3 0BP, UK; stuncay-cagatay@brookes.ac.uk (S.T.C.); amayah@brookes.ac.uk (A.M.); mkadhim@brookes.ac.uk (M.K.); 4 FOCAS Research Institute, Technological University Dublin (TU Dublin), D07 EWV4 Dublin, Ireland; fiona.lyng@tudublin.ie; 5 Helmholtz Zentrum München, German Research Center for Environmental Health GmbH (HMGU), Institute of Radiation Biology, 85764, Neuherberg, Germany; vontoerne@helmholtz-muenchen.de (C.v.T.); zohaib.khan@helmholtz-muenchen.de (Z.N.K.); prabal.subedi@helmholtz-muenchen.de (P.S.); soile.tapio@helmholtz-muenchen.de (S.T.)

**Keywords:** exosomes, miRNome, proteomics, ionizing radiation, neonatal cerebellum, apoptosis

## Abstract

Cell communication via exosomes is capable of influencing cell fate in stress situations such as exposure to ionizing radiation. In vitro and in vivo studies have shown that exosomes might play a role in out-of-target radiation effects by carrying molecular signaling mediators of radiation damage, as well as opposite protective functions resulting in resistance to radiotherapy. However, a global understanding of exosomes and their radiation-induced regulation, especially within the context of an intact mammalian organism, has been lacking. In this in vivo study, we demonstrate that, compared to sham-irradiated (SI) mice, a distinct pattern of proteins and miRNAs is found packaged into circulating plasma exosomes after whole-body and partial-body irradiation (WBI and PBI) with 2 Gy X-rays. A high number of deregulated proteins (59% of WBI and 67% of PBI) was found in the exosomes of irradiated mice. In total, 57 and 13 miRNAs were deregulated in WBI and PBI groups, respectively, suggesting that the miRNA cargo is influenced by the tissue volume exposed to radiation. In addition, five miRNAs (miR-99b-3p, miR-200a-3p, miR-200a, miR-182-5p, miR-182) were commonly overexpressed in the exosomes from the WBI and PBI groups. In this study, particular emphasis was also given to the determination of the in vivo effect of exosome transfer by intracranial injection in the highly radiosensitive neonatal cerebellum at postnatal day 3. In accordance with a major overall anti-apoptotic function of the commonly deregulated miRNAs, here, we report that exosomes from the plasma of irradiated mice, especially in the case of WBI, prevent radiation-induced apoptosis, thus holding promise for exosome-based future therapeutic applications against radiation injury.

## 1. Introduction

Evidence accumulated over the past two decades has indicated that the effects of ionizing radiation are not restricted to irradiated cells but also to non-irradiated bystander or even distant cells manifesting various biological effects. These phenomena have been termed “non-(DNA)-targeted” [1] and include radiation-induced bystander effects [2], genomic instability [3,4], adaptive response [5], low-dose hyper-radiosensitivity [6], delayed reproductive death and induction of genes by radiation [7]. All of these biological effects indicate the involvement of molecules not directly targeted by radiation. 

Exosomes, a subtype of extracellular vesicles (EV), are released from cells into the extracellular space and are capable of transporting cargo (e.g., DNA, RNA, and proteins) between cells as a form of intercellular communication [8,9,10]. MiRNAs are the most abundant cargo in the exosome [11]. They are endogenous non-coding RNA molecules approximately 19–22 nt-long, and important regulators of the protein expression. Proteins, RNA, and other molecules are not randomly loaded into EVs. On the contrary, specialized mechanisms act to ensure a specific cargo, which will define the outcome of communication between the EV producer and the recipient cell. Although the molecular mechanisms that regulate the loading of proteins into EVs have been studied for years, the sorting mechanism of the cargo has been elusive until recently.

EVs including exosomes are secreted by a wide range of cells including immune, neuronal, and cancer cells [12,13,14,15,16]. They are released into the extracellular space and can be found in body fluids such as blood. Of note, the molecular content of exosomes and the biological function that they perform is influenced by the cell type of origin [17]. Circulating blood-derived EVs, secreted from multiple types of cells, offer advantages compared to single cell type-derived EVs in that they may more faithfully recapitulate the complexity of their functions in physiological and pathological conditions. 

Recent reports have indicated that the multiple roles of EVs are largely dependent on the physiological state of the donor cell [18,19,20,21]. Environmental challenges, such as activation or stress conditions, are known to modulate the composition, biogenesis, and secretion of EVs. Stress situations such as hypoxia, starvation, or oxidative stress can alter the exosome content, and thus the message they carry [22].

Exposure to ionizing radiation is among the well-established extracellular factors known to affect EV-based cellular communications, although the mechanisms of this phenomenon are still far from being understood. Previous studies have shown that EV secretion is increased with ionizing radiation exposure in a time- and dose-dependent manner [23,24,25]. While the therapeutic capabilities of exosomes against radiation damage are being studied [26,27,28,29,30,31], growing evidence supports exosomes as key players in radiation-induced bystander effects [32,33,34]. In particular, exosome-mediated miRNA transfer has been shown to play important roles in bystander effects, and exosomes isolated from irradiated conditioned medium have been shown to induce these effects [35]. Nevertheless, there is a big gap in understanding how radiation-induced changes in the composition of exosomes translate into their functional importance. 

In this study, to delineate the radiation-induced changes in miRNAs and protein cargo, we established the microRNA and proteome profiles of plasma exosomes collected from mice that had been sham- (SI), whole-body- (WBI), or partial-body irradiated (PBI) in the lower third of the body with a clinically relevant dose of 2 Gy X-rays 24 h earlier. Spatial variations in dose delivery are relevant for humans as radiation exposure scenario including different PBI patterns are the norm in diagnostic radiology, radiation therapy, and occupational exposures and may have significant implications for human health at low- and intermediate-radiation doses [36,37,38,39]. 

Furthermore, to understand how radiation-induced changes in the miRNA and protein exosomes cargo influence the stress response of distant cells by propagating either damaging or protective signals, we applied an in vivo functional assay interrogating the cerebellum of neonatal mice at postnatal day 3 (P3), highly susceptible to radiation injury. In fact, P3 mouse cerebellum is comparable to the human gestational period of 22–23 weeks, a rapid developmental growth phase characterized by extreme radiosensitivity compared to other brain structures [40]. Moreover, bystander DNA double-strand breaks (DSBs) and apoptosis have previously been detected by our group in the shielded cerebellum of PBI mice at P2, indicating transmission of radiation injury from the exposed distal third of the body to the brain [41]. Therefore, to functionally elucidate the pathophysiological changes induced by irradiation in the exosome cargo, we evaluated the levels of apoptosis in the cerebellum of irradiated or unexposed neonatal mice after the injection of WBI, PBI, or SI plasma-derived exosomes. 

### Experimental Design

**Figure 1:**
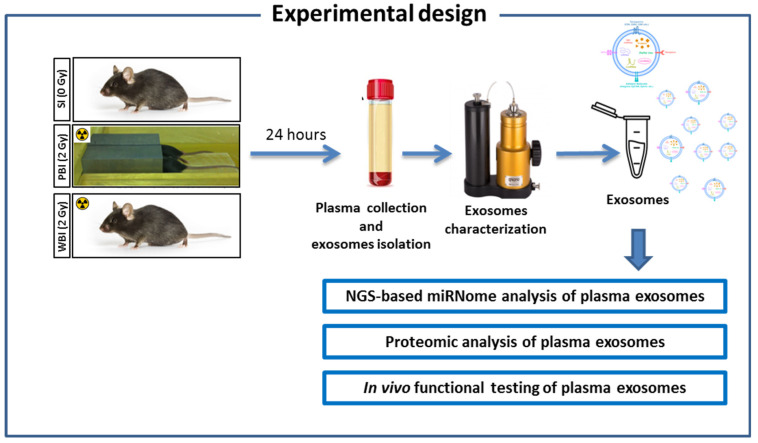
Experimental design scheme. Eight weeks old C57Bl/6 female mice were SI or subjected to WBI or PBI with 2.0 Gy of X-rays. PBI was performed by exposing the lower third of the mouse body, whilst the upper two-thirds were shielded with a lead shield. Twenty-four hours post-irradiation mouse blood was collected and plasma separated by centrifugation for exosome extraction. Exosomes were used for (i) NGS-based miRNome analysis, (ii) proteomic analysis, and (iii) in vivo functional testing.

The experimental design, schematically illustrated in Figure 1, involved C57Bl/6 female mice of eight weeks of age that were SI or subjected to WBI or PBI with 2.0 Gy X-rays. PBI was performed by exposing the lower third of the mouse body, whilst the upper two-thirds were shielded with a shield lead. Twenty-four hours post-irradiation mouse blood was collected and plasma separated by centrifugation for exosome extraction by ultracentrifugation. This time point was selected according to our ex vivo investigations in which, by treating mouse embryonic fibroblasts with the same exosome preparations, we induced changes in DNA damage and calcium, reactive oxygen species, and nitric oxide signalling [25]. Characterization of the exosomal fractions was accomplished using nanoparticle tracking analysis as described earlier [25]. Exosomes were used for (i) NGS-based miRNome analysis, (ii) proteomic analysis of plasma exosomes, and (iii) in vivo functional testing of plasma exosomes.

## 2. Results

### 2.1. MicroRNA Profile of Circulating Plasma Exosomes from 2 Gy WBI and PBI Mice

**Figure 2:**
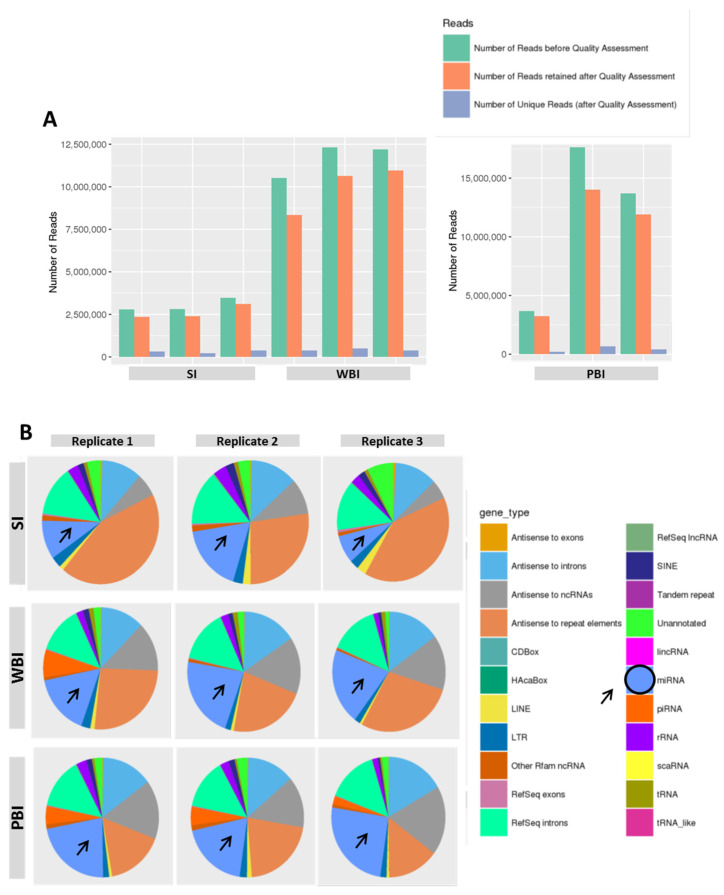
(**A**) Numbers of reads pre- and post-quality filtering and (**B**) characterization of mouse plasma exosomal small non-coding RNA content. The dataset for each treatment group—SI, WBI, and PBI—comprise three individual exosomal preparations from plasma pooled from 5 mice. The fraction of miRNAs is marked by an arrow.

Increasing data demonstrate that miRNAs can influence the way that cells respond to ionizing radiation, making them more radioresistant or radiosensitive through specific pathways. To investigate radiation effects on miRNAs cargo, we performed NGS-based miRNome analysis of plasma exosomes from WBI, PBI, and SI mice at 24 h post-irradiation. The quality check of the samples showed high scores in nearly all the reads (Figure 2A), as their number was substantially unchanged after quality assessment. The pie charts in Figure 2B, illustrating an overview of the distribution of small RNAs in plasma exosomes, indicated an increase in miRNA content in exosomes from WBI and PBI compared to SI mice.

**Figure 3:**
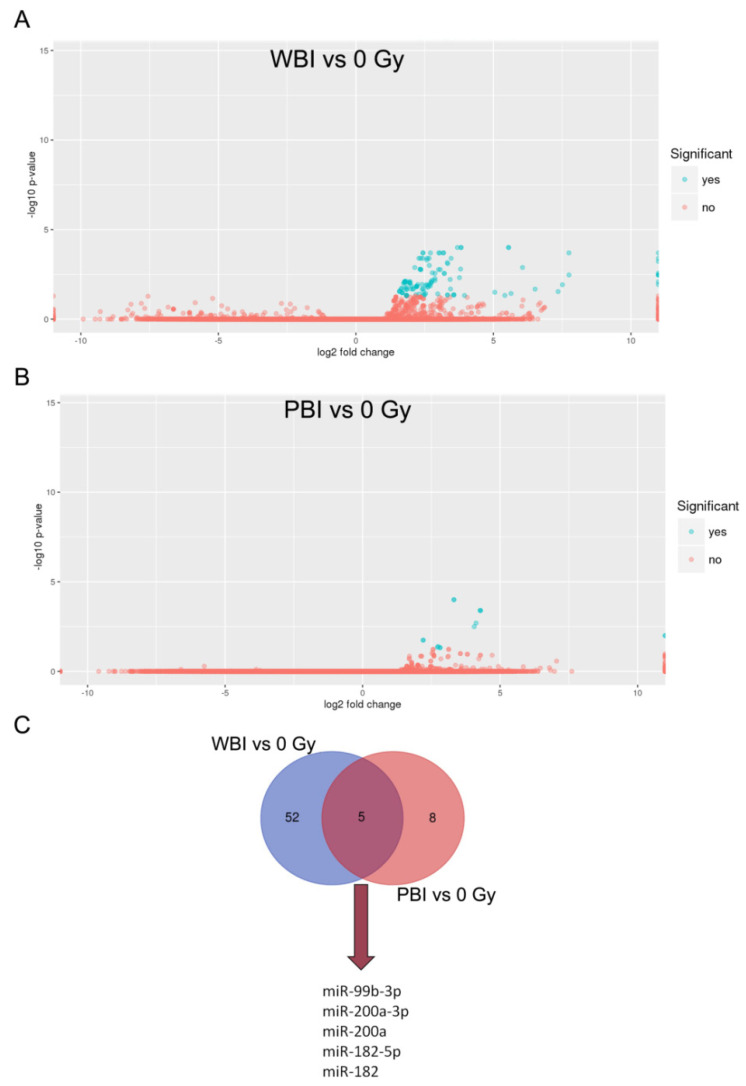
Volcano plots analysis. Fold-change and *p*-values of all differentially expressed miRNAs between 2 Gy-WBI-irradiated (**A**) and 2 Gy-PBI-irradiated (**B**) plasma exosomes vs. 0 Gy plasma exosomes. Fold-change (log2) versus significance (-log10 *p*-value) for each miRNA is shown. Significant miRNAs (FDR < 0.05) are in blue. (**C**) Venn diagram of the significantly deregulated miRNAs in the exosomes of PBI and WBI mice vs. SI mice, with shared miRNAs listed.

Next, we investigated the changes in exosomal miRNA content after WBI and PBI with 2 Gy X-rays in comparison to plasma exosomes of SI mice. MiRNome analysis revealed 57 significantly differentially expressed miRNAs in exosomes from WBI plasma and 13 in exosomes from PBI plasma compared to SI mice. The lower number of deregulated miRNAs in PBI compared to WBI may reflect the contribution of the shielded organs in the exosome composition. Volcano plots show a clear separation between WBI and PBI vs. SI samples (Figure 3A,B). As shown in the Venn diagram (Figure 3C), an overlap in miRNAs expression profiles induced by PBI and WBI was detected with five commonly deregulated miRNAs (miR-99b-3p, miR-200a-3p, miR-200a, miR-182-5p, miR-182). The complete list of the detected miRNA species and the influence of irradiation on their abundance is provided in Supplementary Tables S1 and S2 for WBI and PBI groups, respectively. 

### 2.2. Pathway Analysis of Differentially Regulated miRNAs

**Figure 4:**
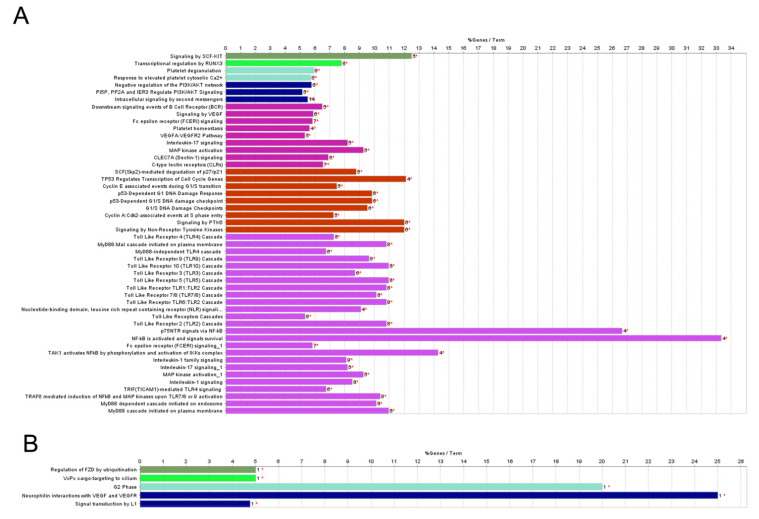
Pathway analyses of plasma-derived exosomal miRNA. Histograms describing the most significant REACTOME pathways associated to the statistically significant miRNAs altered in 2 Gy-WBI (**A**) and in in 2 Gy-PBI (**B**) vs. SI plasma derived exosomes. All differentially expressed miRNAs are listed in Tables S1 and S2. The horizontal axis shows the percentage of pathway deregulation due to differentially expressed genes out of all genes included in each pathway term. At the end of each histogram, the number of differentially expressed genes in the pathway term is reported. Histograms of same color represent groups of terms with interrelations. Red asterisks refer to significance in the percentage of pathway deregulation (* *p* < 0.05).

Next, we looked for biological functions associated with miRNAs that were significantly affected by irradiation. Among over-represented terms showing differences between the sets of SI and WBI-induced miRNA processes, the deregulation of signaling by SCF-kit, by PI3K/AKT, angiogenic signals, and the presence of damage-associated molecular pathways involving DNA damage response/cell cycle regulation and immune responses is predicted (Figure 4A). Instead, pathway analysis for PBI only produced indications of interactions with VEGF and VGFR and regulation of transcription by E2F (Figure 4B).

### 2.3. Proteomic Profiles of Circulating Plasma Exosomes from 2 Gy-Irradiated WBI and PBI Mice 

**Figure 5:**
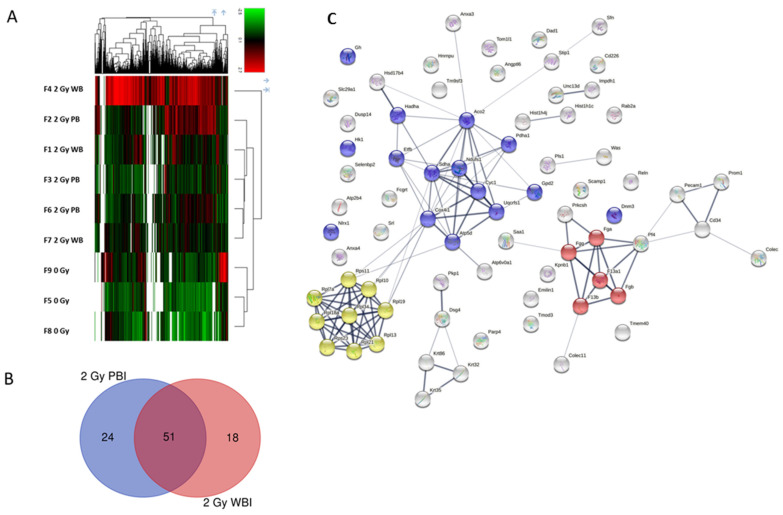
Proteomics analysis of the plasma exosomes 24 h after partial-body irradiation (2 Gy PBI) or whole-body irradiation (2 Gy WBI) compared to the sham-irradiated (SI) controls. (**A**) Supervised heat map showing the separation of PBI and WBI samples from controls based on the significantly differentially expressed proteins in each condition (q > 0.05, fold change > 1.30 or < 0.77, protein identification with at least two unique peptides). The heat map does not discriminate between the WBI and PBI samples. The red bars indicate upregulation and the green bars downregulation. (**B**) Venn diagram showing the number of total and shared significantly differentially regulated proteins using PBI (blue circle) and WBI (red circle) compared to the SI controls. (**C**) Protein interaction analysis showing all significantly differentially regulated proteins in the PBI group. Three clusters are shown: mitochondrial proteins (blue balls), ribosomal proteins (yellow balls), and proteins involved in blood coagulation (red balls). The analysis was performed using the STRING database [42].

The proteomics analysis of the plasma exosomes isolated 24 h after PBI or WBI at 2.0 Gy identified 1001 proteins in total, of which 735 were identified by two unique peptides (Table S3). The supervised heat map analysis of all identified proteins showed a clear separation between SI controls and irradiated samples but did not discriminate between the PBI and WBI samples (Figure 5A). 

This was also seen in the differentially regulated proteins. When compared to the plasma exosomes from SI animals, 75 and 69 proteins using PBI and WBI, respectively, were differentially regulated (Tables S4 and S5). Of these, more than half, namely 51 proteins, were deregulated at both radiation conditions (Figure 5B), and the direction of deregulation was always similar (Tables S4 and S5). 

The protein–protein interaction analysis of the deregulated proteins using PBI showed three protein clusters: mitochondrial proteins, ribosomal proteins, and proteins of the coagulation system (Figure 5C). Similar but smaller clusters were also found using WBI (data not shown). 

The ribosomal and coagulation-associated acute phase proteins were downregulated, whereas most of the mitochondrial proteins were upregulated in the PBI samples compared to SI controls (Table 1). Additionally, cell-adhesion proteins CD34 and platelet endothelial cell adhesion molecule (Pecam1), as well as serum amyloid A-1 protein (Saa1) were upregulated in both radiation conditions (PBI and WBI) (Table 1, Tables S4 and S5). Interestingly, a high number of proteins (67% for PBI and 59% for WBI) were only present in the plasma exosomes from irradiated animals but not in the SI samples (Table 1), indicating that the cargo is highly influenced by irradiation, be it partial- or whole-body exposure.

### 2.4. In Vivo Functional Testing of Plasma-Derived Exosomes on Neonatal Mouse Cerebellum

**Figure 6:**
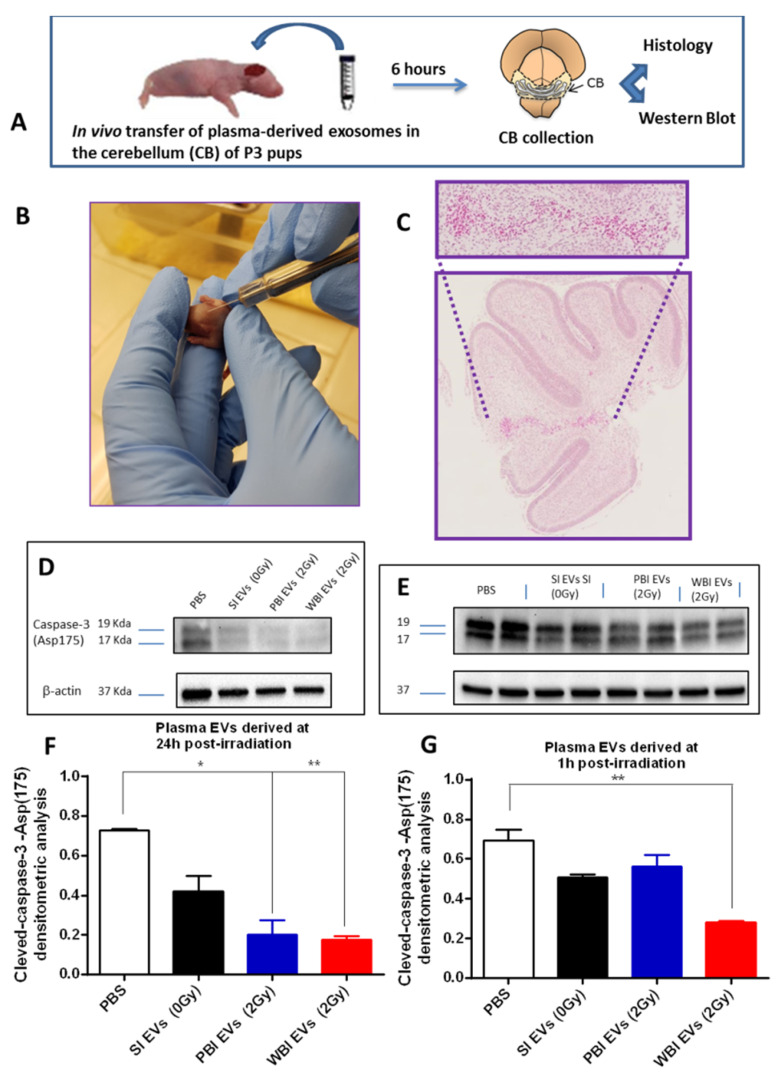
Evaluation of cleaved-caspase-3 response after exosome intracranial injection in unirradiated neonatal mice at P3. (**A**) Experimental design scheme. (**B**) Intracranial injection of exosomes with a Hamilton microsyringe. (**C**) Assessment of injection site after microscopic morphological examination. (**D**,**E**) Lane 1: size marker; lane 2: sham-injected cerebellum (PBS); lane 3: cerebellum injected with 0 Gy-plasma-derived exosomes (EXO 0 Gy); lane 4: cerebellum injected with 2 Gy-PBI-plasma-derived exosomes (EXO PBI); lane5: cerebellum injected with 2 Gy-WBI-plasma-derived exosomes (EXO WBI). Band intensities of cleaved-caspase-3 were sampled three times and normalized for β-actin. (**F**) Densitometric analysis of activated caspase-3 in cerebella injected with plasma exosomes derived at 24 h post-irradiation. (**G**) Densitometric analysis of activated caspase-3 in cerebella injected with plasma Evs derived at 1 h post-irradiation. * *p* < 0.05. ** *p* ≤ 0.01

To enhance our understanding of the role of exosomes in the communication between irradiated and non-irradiated tissues, we evaluated their protective or damaging potential in vivo (Figure 6A). To this aim, we intracranially injected exosomes derived from SI (0 Gy), PBI (2 Gy) or WBI (2 Gy) plasma in the cerebellum of recipient unirradiated neonatal mice (Figure 6B,C). All brain samples were collected at 6 h post-treatment and evaluated for apoptotic response by immunoblotting, using a cleaved-caspase-3 antibody (Figure 6C). We first analyzed the apoptotic level in untreated sham-injected (PBS) P3 cerebellum and compared with that of P3 cerebella injected with plasma exosomes isolated from SI (0 Gy), PBI (2 Gy), and WBI (2 Gy) at 24 h (Figure 6D,F). We detected a nearly significant decrease in the apoptotic level of 42.5% (0.4188 vs. 0.7286, *p* = 0.059) after the injection of exosomes from SI (0 Gy). Statistically significant decreases of 72.5% (0.2004 vs. 0.7286, *p* = 0.0199) and 75.8% (0.1764 vs. 0.7286, *p* = 0.0012) were observed after the injection of plasma exosomes from PBI and WBI mice. Additionally, we investigated the influence of plasma exosomes isolated at an earlier time point of 1 h post-irradiation (Figure 6E,G). A non-significant decrease in the apoptotic level of 26.5% (0.2004 vs. 0.6927, *p* = 0.079) and of 18.8% % (0.5624 vs. 0.6927, *p* = 0.21) was detected after the injection of exosomes from SI (0 Gy) and PBI (2Gy), respectively. Interestingly, a statistically significant decrease of 59.5% (0.2803 vs. 0.6927, *p* = 0.0097) of the cleaved caspase-3 level was observed after the injection of exosomes from WBI (2 Gy), suggesting that plasma exosomes originating from irradiated mice from very early on contain signals preventing cell death (Figure 6E,G). 

**Figure 7:**
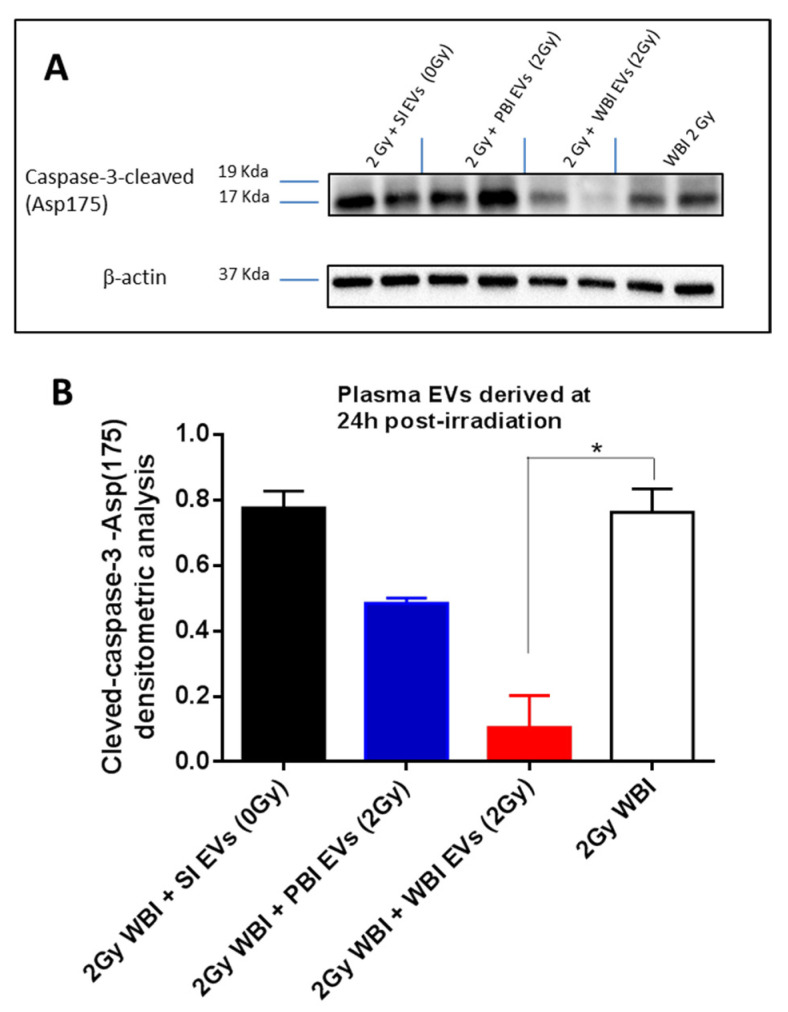
Evaluation of cleaved-caspase-3 response after exosome injection in WBI neonatal mice at P3. (**A**) Lane 1: size marker; lane 2: cerebellum injected with 0 Gy-plasma-derived exosomes (EXO 0 Gy); lane 3: cerebellum injected with 2 Gy-PBI-plasma-derived exosomes (EXO PBI); lane4: cerebellum injected with 2 Gy-WBI-plasma-derived exosomes (EXO WBI), lane 5: cerebellum from 2 Gy whole body irradiated P2 mice. Band intensities of cleaved caspase-3 were sampled three times and normalized for β- actin. (**B**) Densitometric analysis of activated caspase-3 in cerebella irradiated with 2 Gy of X-rays and then injected with plasma exosomes derived at 24 h post-irradiation. * *p* < 0.05.

Finally, to further investigate the possible protective role of exosomes, P3 mice were WBI with 2 Gy (X-ray), and the P3 cerebellum was immediately injected with plasma exosomes isolated from SI (0 Gy), PBI (2 Gy), or WBI (2 Gy) 24 h post-irradiation (Figure 7A,B). Compared to the WBI cerebellum, no significant changes in the cleaved-caspase-3 level were detected after the injection of exosomes from SI (0 Gy) (0.7765 vs. 0.7632, *p* = 0.893) or PBI (2 Gy), (0.4851 vs. 0.7632, *p* = 0.063). In contrast, a statistically significant decrease of 86.2% (0.1055 vs. 0.7632, *p* = 0.032) was observed after the injection of exosomes from WBI (2 Gy), indicative of the capability of plasma exosomes from WBI mice to protect against radiation-induced apoptosis.

## 3. Discussion

The role of exosomes in radiation response has gained increasing attention in recent years. Since exosomes can travel to distant sites due to their release in the blood circulation, they have been suggested as mediators of radiation-induced not-targeted effects [34]. However, this suggestion mostly relies on data derived from purely in vitro experiments and therefore cannot be directly extrapolated to the in vivo context. 

By using an in vivo approach, we have demonstrated here that both the miRNA and protein cargo of circulating plasma exosomes are significantly affected by radiation exposure to a dose of 2 Gy, often corresponding to conventional daily fractions during radiation therapy. Furthermore, exosomes from irradiated mice modulate the level of apoptosis in the cerebellum of non-irradiated and irradiated neonatal mice by exerting a robust protective anti-apoptotic effect.

Exosomes are released from cells in every tissue type [43,44], and their secretion is increased by irradiation in a time- and dose-dependent manner [23,24,25,33,34,45]. Furthermore, as shown by our data on protein and NGS-based miRNA profiling of plasma exosomes, both PBI and WBI influence the exosomal cargo and thereby the radiation response in directly irradiated but also in distant, non-irradiated parts of the body. In addition, the cargo is strongly influenced by the volume of radiation exposed tissue. This is particularly the case in the miRNA content, while the majority of the deregulated proteins were common to WBI and PBI exosomes. 

Of the five commonly differentially expressed miRNAs in the WBI and PBI groups, miR-99 shows the highest degree of deregulation. The expression of this miRNA has already been shown to be radiation responsive [46]. It is involved in the control of DNA damage response by regulating the expression of SNF2H that in turns influences the level of BRACA1 [46].

Additionally, miR-182 is among the commonly deregulated miRNAs in WBI and PBI mice. It is deregulated in the spinal cord of spinal cord injury (SCI) mouse model, and its overexpression has been shown to improve the functional recovery of the SCI mice through the suppression of apoptosis and inflammatory response [47]. The neuroprotective effect of miR-182 in mice following SCI was a consequence of the suppression of apoptosis in neurons [48]. It was also shown that the inhibition of miR-182-5p increased the sensitivity to irradiation in nasopharyngeal carcinoma cells, suggesting that miR-182-5p contributes to cellular radioresistance [49]. Accordingly, miR-182 was upregulated in lung cancer, and its inhibition sensitized cancer cells to ionizing radiation by suppressing cell proliferation and increasing cell apoptosis after irradiation, suggesting that miR-182 might account for radioresistance in lung cancer [50]. 

Components of the miR-200 family, comprising miR-200a, miR-200b, miR-200c, miR-141, and miR-429, are among the most commonly upregulated miRNAs in both WBI and PBI. MiR-141 and miR-200a are upregulated in the WBI group, while miR-411 and miR-429 are in the PBI groups. Most of the miR-200 studies are in the cancer field, where dysregulation of members of this family has been implied in cell growth and tumor progression in several tumor types, while its downregulation generally results in decreased cell proliferation [51,52,53,54]. Recent studies have also shown that reactive oxygen species (ROS) increase the expression of miR-200 from endothelial cells [55]. The MiR-200 group has become an important player in the response to anticancer therapies with special regard to the development of radioresistance [56]. Additionally, all the members of the exosomal miRNAs we found commonly deregulated in WBI and PBI mice have been shown to produce antiapoptotic response in many other experimental settings, also involving radiation exposure.

Irradiation also markedly changed the exosomal protein cargo in both PBI and WBI conditions. Many upregulated proteins were of mitochondrial origin. Mitochondrial proteins have also been found in the EV cargo previously, mainly in cancer cells but also in normal somatic cells [57,58,59]. The function of these proteins in this context is unclear, but mitochondrial components in EVs isolated from human mesenchymal stem cells were able to promote aerobic respiration capacity [60] and may therefore help to functionally resolve radiation-induced tissue injury [61]. Interestingly, mitochondrial components in the EV cargo after pro-inflammatory stimulation such as exposure to lipopolysaccharide have been shown to increase the production of pro-inflammatory cytokines, thereby enhancing inflammation [59,62,63].

We also found upregulation of Pecam1 and Saa1, the major component of the acute phase, in the plasma EV cargo of irradiated mice. Both of these proteins have been indicated in the pro-inflammatory response [48,64]. In contrast, fibrinogens and many ribosomal proteins were downregulated in the exosomal cargo after irradiation. 

A major focus of the present work was to discern whether the radiation-induced modifications in miRNA and protein cargo in circulating exosomes are (i) detrimental, propagating radiation damage; or (ii) protective, promoting repair. We have therefore evaluated the expression of the apoptotic marker caspase-3 after the injection of WBI, PBI, and SI plasma-derived exosomes in the radiosensitive neonatal mouse cerebellum at P3 [41,65]. We focused on the brain for two reasons: first, our previous work provided examples of in vivo out-of-target radiation responses in the brain including induction of DSBs, apoptosis in the cerebellum [41], and miRNome and proteomic changes following radiation exposure of distant tissues in the hippocampus [66]; second, the brain is frequently irradiated in patients with central nervous system (CNS) malignancies, and despite the use of advanced treatment techniques, they often show adverse late outcomes, including cognitive impairments, especially in pediatric patients [67], and neuroprotective intervention is strongly needed. The results of the in vivo functional testing, consistent with the modulation of epigenetic exosomal cargo by irradiation, demonstrated a significant anti-apoptotic role of the WBI- and PBI-derived exosomes in unexposed neonatal cerebellum. However, functional differences between WBI- and PBI-derived exosomes were observed with a stronger protective effect of WBI-derived exosomes, as indicated by the significant anti-apoptotic response in the 2 Gy-irradiated P3 cerebellum that was not observed with PBI exosomes. Furthermore, exosomes from the WBI group isolated at 1 h post-irradiation were able to decrease the apoptotic response in neonatal cerebellum, while PBI-derived exosomes were not. Whether the protective effects of WBI-derived exosomes in the cerebellum are the direct result of miRNA changes or possibly proteins remains uncertain. However, given that the majority of the deregulated proteins were in common between WBI and PBI exosomes, while a higher number of deregulated miRNAs was detected in the WBI compared to the PBI group, our results support a major functional role of miRNAs in mediating the exosome-dependent cell-communication response to radiation exposure.

Significant changes in the miRNAs profile generate subsequent biological effects through the regulation of their target genes. Our bioinformatics analysis of deregulated miRNAs in the WBI group, among the targeted pathway, identified the deregulation of signaling by SCF-kit, by PI3K/AKT, angiogenic signaling, and damage-associated molecular pathways involving DNA damage response/cell cycle regulation and immune responses. Instead, given the lower number of deregulated miRNAs in the PBI group, pathway analysis only produced indications of interactions with VEGF and VGFR and regulation of transcription by E2F. 

Deregulated pathways in the WBI group strongly affect the regulation of inflammation, which may mediate the systemic response to radiation exposure with a potential impact on distant organ sites, and modulation of the DNA damage response. Tissue injury is reported to cause inflammation through the release of damage-associated molecular patterns (DAMPs) which act upon TLR2, TLR4, and TLR9, thereby inducing TLR signaling activation [68]. Numerous investigations report protective effects of TLRs against ionizing radiation. In particular, TLR2, TLR3, TLR4, TLR5, and TLR9 were demonstrated to play a role in the protection against ionizing radiation [69,70,71,72,73]. Our pathway analysis also predicted an activation of NF-kB, an important family of transcription factors controlling inflammatory response, cellular growth, the developmental process, and apoptosis. Many studies report that NF-kB increased the expression in directly irradiated and bystander cells, and the regulation of NF-kB has been shown to be crucial for the bystander response in non-irradiated cells [74]. Interestingly, the miRNA-dependent deregulated pathways in circulating exosomes of WBI mice largely overlapped with the pathways perturbed in the lenses of mice irradiated with 2 Gy -rays 24 h post-irradiation showing a strict interplay between p53 and TLR signaling [75]. 

Similarly, irradiated head and neck cancer cells secreted exosomes within 24 h that increased their survival after irradiation by promoting proliferation and DNA repair efficiency [76]. Similarly, a lower level of apoptosis was observed in irradiated endothelial cells when pre-incubated with EVs from irradiated donors, suggesting an anti-apoptotic function [77]. In accordance with our findings, rat plasma-derived exosomes have also been shown to promote proliferation, migration, and cellular survival in irradiated fibroblasts, improving the healing of cutaneous post-radiation wound when locally injected [78].

Support for our observations also comes from the field of regenerative medicine, where numerous studies have been exploring the potential use of mesenchymal stem cells (MSCs) and MSC exosomes to repair radiation-induced damage in the hematopoietic system, liver, lung, gastrointestinal tract, and skin [79,80,81]. Moreover, EVs have been reported to promote tissue regeneration by creating a pro-regenerative immunomodulatory environment by steering endogenous cells to repair affected tissues and by switching immune responses from pro-inflammatory to tolerogenic in animal studies [82].

An increasing number of studies highlight the contribution of exosomes in the CNS, where, besides being key players in the intercellular communication underlying physiological processes such as synaptic plasticity, they were found to cross the blood–brain barrier in injured CNS, signaling the propagation of neuroinflammation [83,84,85] or supporting neuronal survival under ischemic stress [86,87,88]. In addition, cranial transplantation of stem-cell-derived exosomes has been shown to impart significant neuroprotective effects within the irradiated brain microenvironment [89].

The question of how the contents of the exosomal cargo depends on the nature of donor and recipient cells remains unsolved. Ex vivo investigations in which exosome preparations isolated 24 h post-irradiation from WBI or PBI mouse organs or plasma were incubated with non-irradiated mouse embryonic fibroblasts showed the induction of DNA damage, and calcium, ROS and nitric oxide signaling [25]. In contrast, we show here that similar plasma exosome preparations enhance radioresistance when tested in the unexposed or irradiated mouse cerebellum in vivo. This underlines the difficulties when extrapolating EV function from in vitro to in vivo models. Although we are unable to reconcile this discrepancy, our in vivo findings strongly support a role for plasma exosomes isolated from irradiated mice in ameliorating brain radiation-induced toxicity by preventing the induction of spontaneous and radiation-induced apoptosis in the cerebellum.

Although our group demonstrated the transmission of DNA DSBs, apoptosis, and the induction of oncogenic bystander effects in the neonatal cerebellum following irradiation of the lower third of the mouse body [41,90,91,92], we here show that plasma exosomes from irradiated mice attenuate neuroinflammatory response and protect the cerebellar neurons from radiation-induced cell death. These apparently contradictory results can be reconciled, bearing in mind that the induction of survival mechanisms, mediated by proteomic and miRNome-driven changes, may in turn promote carcinogenesis through the induction of cell-death resistance in cells harboring DNA damage. Of note, these findings also exclude exosome-mediated systems for the transmission of radiation-induced bystander damage, instead implying other mechanisms in the transportation of detrimental signals from the irradiated regions to distant non-irradiated cerebellum. Molecular signals could be transmitted through gap junctions, and/or mediator transfer mechanisms [90,93,94]. Of note, decreased tissue communication by genetic ablation of one copy of Connexin43 reduced the tumor response to radiation in the bystander cerebellum [90].

In summary, WBI and PBI of mice induce changes in miRNA and proteomic profiles of plasma-derived exosomes that mediate systemic responses to radiation exposure and thereby have an impact on distant organ sites. However, while the majority of regulatory functions of exosomes have been delineated using in vitro systems, there is a big gap in understanding how these changes are regulated and translated into their functional importance in vivo. Further experiments are required for a better understanding of exosome biology in vivo and functional testing in different organ systems. This will greatly enhance the future promise for exosomes-based therapeutic applications to eventually reduce radiation side effects in normal tissues.

## 4. Material and Methods

### 4.1. Mouse Irradiation 

C57Bl/6J female mice of 8 weeks of age were subjected to WBI or PBI with 2.0 Gy of X-rays. Irradiation was performed using a Gilardoni CHF 320 G X-ray generator (Gilardoni, Mandello del Lario Italy) operated at 250 kVp, 1 mA for 0.1 Gy and 15 mA for 2.0 Gy, with Half-Value Layer = 1.6 mm Cu (additional filtration of 2.0 mm Al and 0.5 mm Cu). PBI was performed by exposing the lower third of the mouse body, whilst the upper two thirds were shielded with a lead-shield. Additional groups of mice were SI. 

### 4.2. Isolation of Plasma-Derived Exosomes

One or twenty-four hours after SI, PBI, or WBI, animals were sacrificed, blood was collected from the submandibular vein, and blood plasma was separated and snap-frozen for later exosome extraction. All the samples were shipped to Oxford Brookes University (OBU) for exosome isolations and characterization as described previously [25]. RNA was extracted with the Total Exosome RNA and Protein Isolation Kit (cat 4478545, Invitrogen) according to the manufacturer’s instructions. 

### 4.3. MicroRNA Profiling from Plasma-Derived Exosomes

To profile miRNAs, exosomal RNA preparations from three biological replicates, each consisting of pooled plasma from five mice, were used for each treatment group (n = 15 mice/group) to identify differences between the groups and to avoid individual variances between samples. RNA samples were mailed to System Biosciences (SBI) for RNA-seq analysis utilizing SBI’s Exosome RNA NGS Service (https://www.systembio.com/services/exosome-services/exo-ngs, accessed on 18 January 2022). Briefly, Illumina NGS libraries were prepared and sequenced using an Illumina HiSeq2000 sequence analyzer (Illumina Inc., San Diego, CA, USA). Data analysis occurred using the Maverix Exosome RNA-seq Analysis platform (Maverix Biomics Inc., San Mateo, CA). The web-based analysis service included library sequence quality control metrics, normalization of raw sequence read, data analysis for relative RNA abundance, and identity and differential expression analysis. Differentially expressed plasma exosome RNAs were reported as log2 fold change with significance considered at *p* < 0.05. 

### 4.4. Pathway Analysis of Plasma-Derived Exosomal miRNA

Statistically significant miRNAs (*p* < 0.05, log fold-change > 1.5) were used for gene/miRNA enrichment analysis with Cytoscape plug-in ‘‘CluePedia’’ (version 1.1.7) and “ClueGo” (version 2.1.7) [95]. The pathway analysis was obtained selecting the genes with a validated miRTarBase (score 0.6) with an enrichment over the 20 top target genes for each miRNA of the list. The final enriched list was then selected to identify the affected pathways into the REACTOME database (https://reactome.org, accessed on 18 January 2022), considering a minimum number of genes into the pathway equal to 4 and 2 for WBI vs. SI and PBI vs. SI lists, respectively, and with a percentage not less than 5 or 4 for the two conditions, respectively.

### 4.5. Mass Spectrometry (MS) Sample Preparation and Measurement for Protein Profiling of Plasma-Derived Exosomes 

The proteomics analysis was performed using three biological samples, each pooled from plasma EVs isolated from 10 mice in order to reach sufficient amount of material. Measures of 10 µg of sample were enzymatically digested using a modified filter-aided sample preparation (FASP) protocol as described previously [96,97]. Peptides were stored at –20 °C until MS measurement.

MS measurement was performed in the data-dependent acquisition (DDA) mode. MS data were acquired on a Q Exactive (QE) high-field (HF) mass spectrometer (Thermo Fisher Scientific Inc. Waltham, MA, USA) as described [98]. 

### 4.6. Data Processing—Protein Identification

Proteome Discoverer 2.4 software (Thermo Fisher Scientific; version 2.4.1.15) was used for peptide and protein identification via a database search (Sequest HT search engine) against Swiss-Prot mouse database (Release 2020_02, 17061 sequences), considering full tryptic specificity, allowing for up to one missed tryptic cleavage site, precursor mass tolerance 10 ppm, fragment mass tolerance 0.02 Da. Carbamidomethylation of Cys was set as a static modification. Dynamic modifications included deamidation of Asn and Gln, oxidation of Met, and a combination of Met loss with acetylation on protein N-terminus. Percolator was used for validating peptide spectrum matches and peptides, accepting only the top-scoring hit for each spectrum, and satisfying the cutoff values for FDR <1%, and posterior error probability <0.01. The final list of proteins complied with the strict parsimony principle.

### 4.7. Data Processing—Label-Free Quantification

The quantification of proteins was based on the area value of the abundance values for unique plus razor peptides. Abundance values were normalized in a retention-time-dependent manner to account for sample-loading errors. The protein abundances were calculated summing up the abundance values for admissible peptides. The final abundance ratio (fold change) was calculated using median abundance values of three replicate analyses each. The statistical significance of the ratio change was ascertained employing the T-test approach described in Navarro et al. [99] which is based on the presumption that we look for expression changes for proteins that are just a few in comparison to the number of total proteins being quantified. The quantification variability of the non-changing “background” proteins can be used to infer which proteins change their expression in a statistically significant manner. Proteins were defined as significantly up- or downregulated if they fulfilled the following filtering criteria: fold change of ≥1.30 (upregulation) or ≤0.77 (downregulation), (ii) q (FDR corrected *p*) ≤0.05, and (iii) protein identification by at least two unique peptides.

### 4.8. Data Availability

The mass spectrometry proteomics data have been deposited to the ProteomeXchange Consortium via the PRIDE [100] partner repository with the dataset identifier PXD026408.

### 4.9. In Vivo Exosomes Transfer

Exosomes isolated from plasma of animals SI, PBI, and WBI after 2 Gy of X-rays suspended in PBS were intracranially injected in the brain of P3 unirradiated or 2 Gy-WBI neonatal mice, through a Hamilton microsyringe, using 4 μL per mice (5 − 12.5 × 107/μL). Mice were sacrificed at 6 h post-injection or post-irradiation and brain tissue was snap-frozen or collected for histology.

### 4.10. Histological Analysis 

After exosome injection, to assess the injection site, cerebella of C57Bl/6 pups (n = 3) were processed for histology by standard techniques, and cerebella sections were cut (4 μm) for H&E staining and microscopic morphological examination.

### 4.11. Western Blot

For immunoblot, total proteins were extracted from cerebella (n = 2) at P3. Experimental groups included sham-injected (PBS) mice; mice injected with 0 Gy-plasma-derived exosomes (Evs SI (0 Gy); mice injected with 2 Gy PBI-plasma-derived exosomes (Evs PBI (2 Gy); mice injected with 2 Gy WBI-plasma-derived exosomes (Evs WBI (2 Gy); 2 Gy whole-body-irradiated mice (2 Gy WBI). All brain samples were collected at 6 h post-treatment. Proteins were extracted, normalized, separated, and immunoblotted as described [76]. To evaluate apoptosis, samples were incubated with Cleaved-caspase-3 (Asp 175) rabbit polyclonal antibody (dilution 1:1000; Cell Signaling Technology, Danvers, MA, USA). Monoclonal antibody against β-actin (dilution 1:10,000, Sigma-Aldrich, St. Louis, MO, USA) was used as a loading control. Specific proteins were visualized with ChemiDoc system XRS+ Biorad and quantified using ImageJ software. 

### 4.12. Statistical Analysis

qPCR and Western blot analyses are reported as means ± standard error of the means (SEM), and a *t*-test was used for the determination of statistical difference between groups; *p* ≤ 0.05 was considered statistically significant. Analyses were performed using GraphPad Prism 5.0 (GraphPad Software, San Diego, CA, USA).

## 5. Conclusions

Using neonatal mouse cerebellum, our group demonstrated in vivo the anti-apoptotic potential of circulating exosomes derived from the plasma of irradiated mice. This response was mediated by changes in the exosome cargo, affecting both miRNA and protein contents and modulating damage-associated molecular pathways, immunoresponse, and angiogenesis. Notably, we show here that exosomes are important in the context of radiation-injured CNS and prove the relevance of exosomal-mediated neuroprotection in vivo. Such knowledge may pave the road for therapeutic applications of exosomes to the treatment of radiation injury in the CNS, including the resolution of normal tissue toxicities associated with radiotherapy, although further basic research and technical development will be required.

## Figures and Tables

**Table 1:** Significantly differentially regulated proteins in mouse blood exosomes of PBI at 2 Gy.

Accession	Description	Gene Symbol	Abundance Ratio (2 Gy, PBI)/0 Gy)
Q9D0M3	**Cytochrome c1, heme protein, mitochondrial**	*Cyc1*	0.010
O54990	**Prominin-1**	*Prom1*	0.010
Q8JZM8	**Mucin-4**	*Muc4*	0.010
Q62168	**Keratin, type I cuticular Ha2**	*Krt32*	0.010
Q497I4	**Keratin, type I cuticular Ha5**	*Krt35*	0.010
O70456	**14-3-3 protein sigma**	*Sfn*	0.010
P97350	Plakophilin-1	*Pkp1*	0.010
Q8BZ98	Dynamin-3	*Dnm3*	0.010
P70315	Wiskott–Aldrich syndrome protein homolog	*Was*	0.010
Q923U0	TOM1-like protein 1	*Tom1l1*	0.010
P47963	60S ribosomal protein L13	*Rpl13*	0.010
P12970	60S ribosomal protein L7a	*Rpl7a*	0.010
P62281	40S ribosomal protein S11	*Rps11*	0.010
O09167	**60S ribosomal protein L21**	*Rpl21*	0.010
P62717	**60S ribosomal protein L18a**	*Rpl18a*	0.010
Q8VCM7	**Fibrinogen gamma chain**	*Fgg*	0.031
Q8K0E8	Fibrinogen beta chain	*Fgb*	0.038
E9PV24	**Fibrinogen alpha chain**	*Fga*	0.040
Q9D3D9	ATP synthase subunit delta, mitochondrial	*Atp5d*	0.040
Q7TMD7	**Desmoglein-4**	*Dsg4*	0.094
Q99K41	EMILIN-1	*Emilin1*	0.096
P97861	**Keratin, type II cuticular Hb6**	*Krt86*	0.106
Q60841	**Reelin**	*Reln*	0.139
Q9JHJ0	**Tropomodulin-3**	*Tmod3*	0.143
Q3SXB8	Collectin-11	*Colec11*	0.143
Q8BH61	**Coagulation factor XIII A chain**	*F13a1*	0.151
Q8R0Z6	Angiopoietin-related protein 6	*Angptl6*	0.154
P15864	**Histone H1.2**	*Hist1h1c*	0.168
Q9JLY7	**Dual specificity protein phosphatase 14**	*Dusp14*	0.180
Q63836	Selenium-binding protein 2	*Selenbp2*	0.184
Q8CF98	Collectin-10	*Colec10*	0.184
P62806	Histone H4	*Hist1h4j*	0.187
Q9D1R9	60S ribosomal protein L34	*Rpl34*	0.189
P84099	60S ribosomal protein L19	*Rpl19*	0.201
Q07968	Coagulation factor XIII B chain	*F13b*	0.204
Q6ZWV3	60S ribosomal protein L10	*Rpl10*	0.210
P62267	40S ribosomal protein S23	*Rps23*	0.244
P17710	Hexokinase-1	*Hk1*	5.598
P19783	**Cytochrome c oxidase subunit 4 isoform 1, mitochondrial**	*Cox4i1*	6.534
Q64521	**Glycerol-3-phosphate dehydrogenase, mitochondrial**	*Gpd2*	6.570
Q64314	**Hematopoietic progenitor cell antigen CD34**	*Cd34*	8.780
Q9Z126	Platelet factor 4	*Pf4*	14.195
Q9DCW4	**Electron transfer flavoprotein subunit beta**	*Etfb*	100.00
Q8K021	**Secretory carrier-associated membrane protein 1**	*Scamp1*	100.00
Q4FJU9	**Transmembrane protein 40**	*Tmem40*	100.00
P70168	**Importin subunit beta-1**	*Kpnb1*	100.00
Q61559	**IgG receptor FcRn large subunit p51**	*Fcgrt*	100.00
Q8VEK3	**Heterogeneous nuclear ribonucleoprotein U**	*Hnrnpu*	100.00
B2RUP2	**Protein unc-13 homolog D**	*Unc13d*	100.00
Q7TQ48	**Sarcalumenin**	*Srl*	100.00
P51660	**Peroxisomal multifunctional enzyme type 2**	*Hsd17b4*	100.00
P53994	**Ras-related protein Rab-2A**	*Rab2a*	100.00
Q91VD9	**NADH-ubiquinone oxidoreductase 75 kDa subunit, mitochondrial**	*Ndufs1*	100.00
Q8BMS1	**Trifunctional enzyme subunit alpha, mitochondrial**	*Hadha*	100.00
P06880	**Somatotropin**	*Gh*	100.00
Q6Q477	**Plasma membrane calcium-transporting ATPase 4**	*Atp2b4*	100.00
O35639	**Annexin A3**	*Anxa3*	100.00
Q3TL44	**NLR family member X1**	*Nlrx1*	100.00
Q9JIM1	**Equilibrative nucleoside transporter 1**	*Slc29a1*	100.00
P35486	**Pyruvate dehydrogenase E1 component subunit alpha, mitochondrial**	*Pdha1*	100.00
O08795	**Glucosidase 2 subunit beta**	*Prkcsh*	100.00
Q9ET30	**Transmembrane 9 superfamily member 3**	*Tm9sf3*	100.00
Q8K2B3	**Succinate dehydrogenase [ubiquinone] flavoprotein subunit, mitochondrial**	*Sdha*	100.00
P50096	**Inosine-5’-monophosphate dehydrogenase 1**	*Impdh1*	100.00
Q99KI0	**Aconitate hydratase, mitochondrial**	*Aco2*	100.00
P97429	**Annexin A4**	*Anxa4*	100.00
Q9Z1G4	**V-type proton ATPase 116 kDa subunit a isoform 1**	*Atp6v0a1*	100.00
Q08481	**Platelet endothelial cell adhesion molecule**	*Pecam1*	100.00
Q3V0K9	**Plastin-1**	*Pls1*	100.00
E9PYK3	**Protein mono-ADP-ribosyltransferase PARP4**	*Parp4*	100.00
Q9CR68	**Cytochrome b-c1 complex subunit Rieske, mitochondrial**	*Uqcrfs1*	100.00
P61804	**Dolichyl-diphosphooligosaccharide–protein glycosyltransferase subunit DAD1**	*Dad1*	100.00
P05366	**Serum amyloid A-1 protein**	*Saa1*	100.00
Q60864	Stress-induced-phosphoprotein 1	*Stip1*	100.00
Q8K4F0	CD226 antigen	*Cd226*	100.00

^1^ Protein accession number, description, gene symbol, and abundance ratio (fold change) are shown. The deregulated proteins shared between PBI and WBI are shown in bold. Abundance ratios of 100.00 indicate that the protein is only found in the irradiated sample.

## Data Availability

Other datasets analyzed during the study are available from the corresponding authors on reasonable request.

## Supplementary Materials

The following supporting information can be downloaded at: https://www.mdpi.com/article/10.3390/ijms23042169/s1.
