# Supplementary material for: Micro-RNA and Proteomic Profiles of Plasma-Derived Exosomes from Irradiated Mice Reveal Molecular Changes Preventing Apoptosis in Neonatal Cerebellum
Source: Int J Mol Sci. 2022 Feb 16;23(4):2169. doi: 10.3390/ijms23042169 (PMC8878539; doi:10.3390/ijms23042169)
Supplement: Supplementary file 1 [file ijms-23-02169-s001.zip › Tables S1 and S2.pptx]

## Slide 1
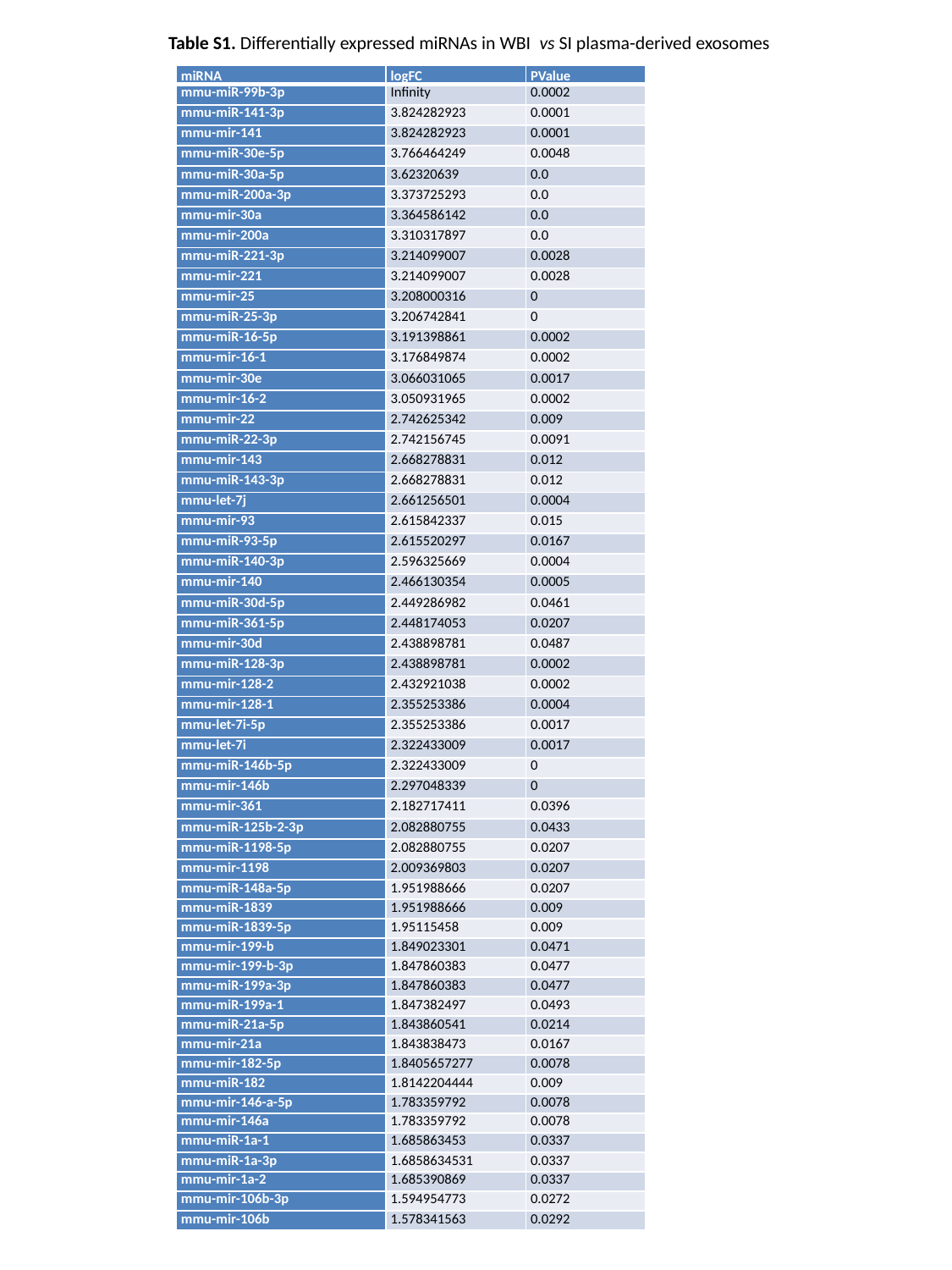

Table S1. Differentially expressed miRNAs in WBI vs SI plasma-derived exosomes
| miRNA | logFC | PValue |
| --- | --- | --- |
| mmu-miR-99b-3p | Infinity | 0.0002 |
| mmu-miR-141-3p | 3.824282923 | 0.0001 |
| mmu-mir-141 | 3.824282923 | 0.0001 |
| mmu-miR-30e-5p | 3.766464249 | 0.0048 |
| mmu-miR-30a-5p | 3.62320639 | 0.0 |
| mmu-miR-200a-3p | 3.373725293 | 0.0 |
| mmu-mir-30a | 3.364586142 | 0.0 |
| mmu-mir-200a | 3.310317897 | 0.0 |
| mmu-miR-221-3p | 3.214099007 | 0.0028 |
| mmu-mir-221 | 3.214099007 | 0.0028 |
| mmu-mir-25 | 3.208000316 | 0 |
| mmu-miR-25-3p | 3.206742841 | 0 |
| mmu-miR-16-5p | 3.191398861 | 0.0002 |
| mmu-mir-16-1 | 3.176849874 | 0.0002 |
| mmu-mir-30e | 3.066031065 | 0.0017 |
| mmu-mir-16-2 | 3.050931965 | 0.0002 |
| mmu-mir-22 | 2.742625342 | 0.009 |
| mmu-miR-22-3p | 2.742156745 | 0.0091 |
| mmu-mir-143 | 2.668278831 | 0.012 |
| mmu-miR-143-3p | 2.668278831 | 0.012 |
| mmu-let-7j | 2.661256501 | 0.0004 |
| mmu-mir-93 | 2.615842337 | 0.015 |
| mmu-miR-93-5p | 2.615520297 | 0.0167 |
| mmu-miR-140-3p | 2.596325669 | 0.0004 |
| mmu-mir-140 | 2.466130354 | 0.0005 |
| mmu-miR-30d-5p | 2.449286982 | 0.0461 |
| mmu-miR-361-5p | 2.448174053 | 0.0207 |
| mmu-mir-30d | 2.438898781 | 0.0487 |
| mmu-miR-128-3p | 2.438898781 | 0.0002 |
| mmu-mir-128-2 | 2.432921038 | 0.0002 |
| mmu-mir-128-1 | 2.355253386 | 0.0004 |
| mmu-let-7i-5p | 2.355253386 | 0.0017 |
| mmu-let-7i | 2.322433009 | 0.0017 |
| mmu-miR-146b-5p | 2.322433009 | 0 |
| mmu-mir-146b | 2.297048339 | 0 |
| mmu-mir-361 | 2.182717411 | 0.0396 |
| mmu-miR-125b-2-3p | 2.082880755 | 0.0433 |
| mmu-miR-1198-5p | 2.082880755 | 0.0207 |
| mmu-mir-1198 | 2.009369803 | 0.0207 |
| mmu-miR-148a-5p | 1.951988666 | 0.0207 |
| mmu-miR-1839 | 1.951988666 | 0.009 |
| mmu-miR-1839-5p | 1.95115458 | 0.009 |
| mmu-mir-199-b | 1.849023301 | 0.0471 |
| mmu-mir-199-b-3p | 1.847860383 | 0.0477 |
| mmu-miR-199a-3p | 1.847860383 | 0.0477 |
| mmu-miR-199a-1 | 1.847382497 | 0.0493 |
| mmu-miR-21a-5p | 1.843860541 | 0.0214 |
| mmu-mir-21a | 1.843838473 | 0.0167 |
| mmu-mir-182-5p | 1.8405657277 | 0.0078 |
| mmu-miR-182 | 1.8142204444 | 0.009 |
| mmu-mir-146-a-5p | 1.783359792 | 0.0078 |
| mmu-mir-146a | 1.783359792 | 0.0078 |
| mmu-miR-1a-1 | 1.685863453 | 0.0337 |
| mmu-miR-1a-3p | 1.6858634531 | 0.0337 |
| mmu-mir-1a-2 | 1.685390869 | 0.0337 |
| mmu-mir-106b-3p | 1.594954773 | 0.0272 |
| mmu-mir-106b | 1.578341563 | 0.0292 |

## Slide 2
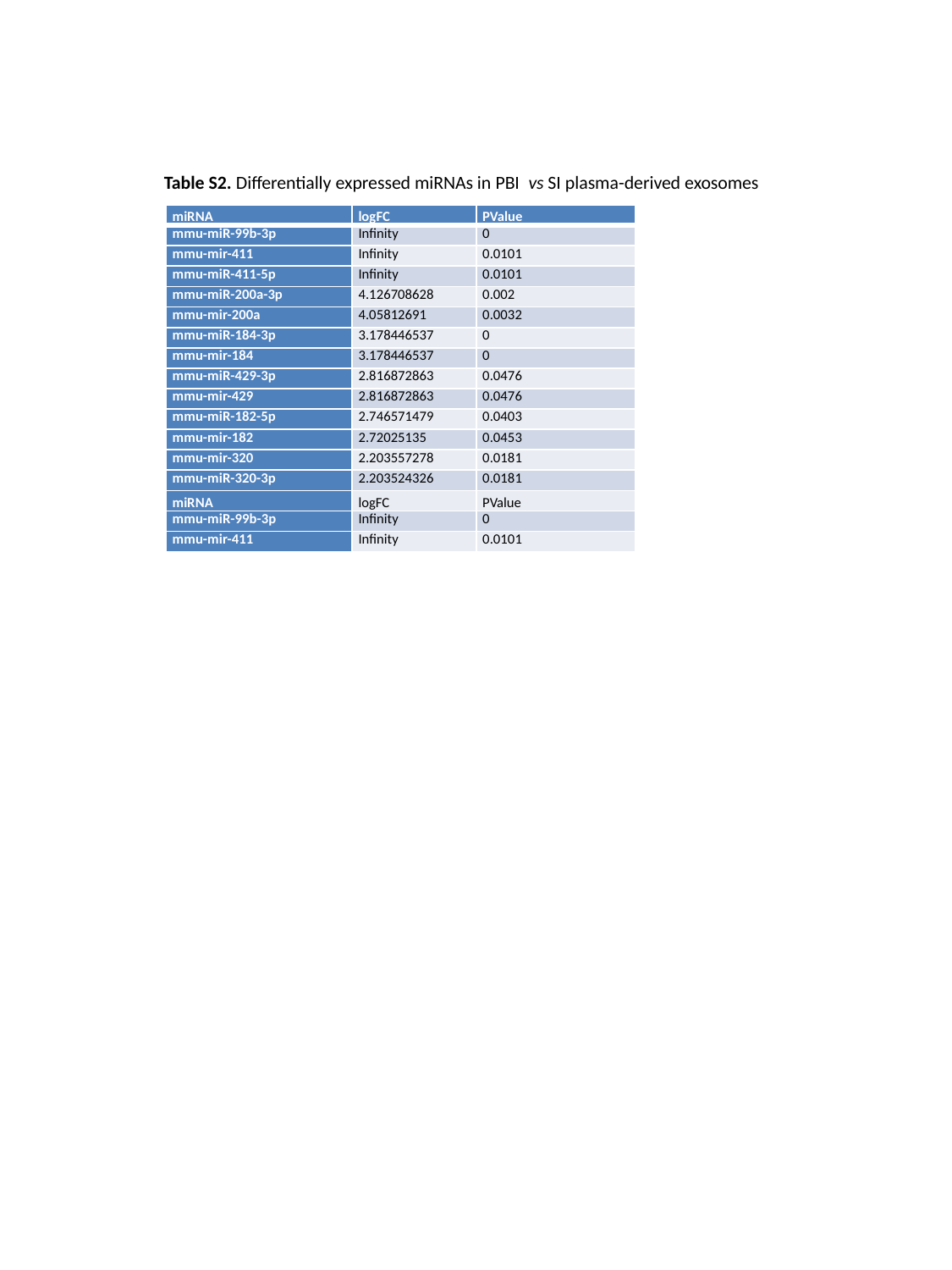

Table S2. Differentially expressed miRNAs in PBI vs SI plasma-derived exosomes
| miRNA | logFC | PValue |
| --- | --- | --- |
| mmu-miR-99b-3p | Infinity | 0 |
| mmu-mir-411 | Infinity | 0.0101 |
| mmu-miR-411-5p | Infinity | 0.0101 |
| mmu-miR-200a-3p | 4.126708628 | 0.002 |
| mmu-mir-200a | 4.05812691 | 0.0032 |
| mmu-miR-184-3p | 3.178446537 | 0 |
| mmu-mir-184 | 3.178446537 | 0 |
| mmu-miR-429-3p | 2.816872863 | 0.0476 |
| mmu-mir-429 | 2.816872863 | 0.0476 |
| mmu-miR-182-5p | 2.746571479 | 0.0403 |
| mmu-mir-182 | 2.72025135 | 0.0453 |
| mmu-mir-320 | 2.203557278 | 0.0181 |
| mmu-miR-320-3p | 2.203524326 | 0.0181 |
| miRNA | logFC | PValue |
| mmu-miR-99b-3p | Infinity | 0 |
| mmu-mir-411 | Infinity | 0.0101 |
